# Changes of Noradrenaline in Brain Homogenate of Rats with Brain Injury Secondary to Intracerebral Hemorrhage: A Study of the Mechanism

**Published:** 2005-06

**Authors:** Zhi-Qiang Xu, Hua-Dong Zhou, Xiao-Jiang Jiang, Jing-Zhou Wang, Man-E Chen

**Affiliations:** *Third Department of Neurology, Daping Hospital, Research Institute of Surgery, Third Military Medical University, Chongqing, China*

**Keywords:** cerebral hemorrhage, noradrenaline, brain injury

## Abstract

**Objective:**

To observe the changes of noradrenaline (NE) content dynamically in the homogenate of rat brain tissues during experimental intracerebral hemorrhage (ICH), so as to understand the role of NE in secondary brain injury.

**Methods:**

Seventy Wistar rats were randomly assigned into sham operation group and ICH group, each group subdivided into different time phase points as pre-operation, 0.5, 6, 12, 24, 48 and 72 h post-operation groups (n=5). ICH model was established by injection of collagenase and heparin into rat caudate nucleus, and the changes of NE content in the peripheral tissues of the hematoma, hypothalamus and brainstem were observed respectively at following time points as before operation and 0.5, 6, 12, 48 and 72 h after the operation. NE was determined by high-performance liquid chromatography.

**Results:**

NE activities in the peripheral tissues of the hematoma, hypothalamus and brainstem increased synchronously 0.5 h after operation, peaked at 24 h, and then began to decline at 48 h. At the same time, the neurobehavioral score varied synchronously together with NE.

**Conclusion:**

NE is involved in the pathogenesis of secondary damage of the brain during ICH.

## INTRODUCTION

Brain tissue injury secondary to cerebral hemorrhage has been acknowledged as one of the main causes to affect the patient’s prognosis, and has received much attention from researchers in recent years. Currently the physiopathological mechanism of secondary brain tissue injury following cerebral hemorrhage has not been fully clarified, but it is believed that the secondary injury is the result of the interaction of multiple mechanisms, in which monoamine transmitters play an important role. In this study, we observed the changes of noradrenaline (NE) levels dynamically in the peripheral tissues of the hemotoma, hypothalamus and brainstem of rats during cerebral hemorrhage, attempt to understand the implications of the changes.

## MATERIALS AND METHODS

### Animals and reagents

Seventy healthy Wistar rats regardless of genders and weighing 250-300 g were supplied by the Animal Center of Daping Hospital, Third Military Medical University. The rats were randomly assigned into operative group and sham operation group in equal number (n=35), and each group was subdivided into different time phases as before operation, 0.5, 6, 12, 24, 48 and 72 h post-operation, there were 5 rats sacrificed at each time points. Collagenase (VII-S type) and standard NE were manufactured by Sigma Co., USA.

### Methods

**Model establishment.** According to the method described (1), rat models of intracerebral hemorrhage were established by injection with both collagenase and heparin. In brief, the rats were anesthetized with intraperitoneal injection of pentobarbital sodium and fixed in prone position in a stereotaxic apparatus. Through a median incision in the scalp, the periosteum was dissociated with a periosteotome to expose the anterior fontanelle and coronal suture. A hole was drilled with dental drill at the right caudate nucleus as the point for injection of 1 μL saline containing 1 U/μL collagenase and 7 U/μL heparin (AP1/RAT3/DV5, according to the method of Paxinous and Watson). The sham operation group received injection of saline of the same quality instead. The needle was not withdrawn till 5 min after the injection and the scalp was sutured, followed by observation neurological symptoms and signs. The observation was performed at time points as before operation and 30 min, 6, 12, 24, 48, and 72 h after the operation.

**Neurobehavioral scoring.** After the rats waked from anesthesia after operation, their neurological deficit status were assessed according to Zea-Longa’s method (2) and classified into 5 grades as follows: grade 0, in which the rats retained normal activity without neurologic impairment; grade 1, in which the rats failed to fully stretch their forelimbs on the paralytic side when held by the tail; grade 2, in which the rat rotated to the paralytic side; grade 3, in which the rats toppled and fell to the paralytic side; grade 4, in which the rat could not walk spontaneously with conscious disturbance.

**Preparation of the tissue samples.** Immediately after decapitation, the rat brain was collected to separate the hypothalamus and the peripheral tissues of the hematoma on ice (the right basal ganglia was collected in the sham operation group), which were weighed and grinded into homogenate in the presence of 0.1mol/L perchloric acid (20 μl per 1 mg tissue sample). After centrifugation at 18 000 r/min (4°C) for 20 min, the supernatant was collected and stored at -70°C for later use.

**Measurement of NE content in the brain tissue.** NE content in the brain tissues was determined strictly following the method described in the literature (3).

**Statistical analysis.** All data were processed by Microsoft Excel 7.0, and the results expressed as Mean ± SD. Analysis of variance of the results was performed by the authors and means were compared between the groups.

## RESULTS

### The changes of neurobehavioral score

Neurobehavioral score did not vary at the time points in the sham operation group (score=0). It began to rise at 0.5 h after hemorrhage, peak at 24 h after operation. Its level was still higher than that of those before operation till 72 h (*P*<0.01) (Table 1).

**Table 1:** Changes of neurobehavioral score in the two groups (n=5)

Group	Before operation	After operation (h)					
		0.5	6	12	24	48	72

Sham operation	0	0	0	0	0	0	0
Hemorrhage	0	0.58 ± 0.33^a^^b^	2.20 ± 0.56^a^^b^	3.25 ± 0.91^a^^b^	3.60 ± 0.81^a^^b^	3.05 ± 0.73^a^^b^	1.22 ± 0.52^a^^b^

a t= 18.7565-46.5542, *P*<0.01, vs the level before operation;

b t=48.6342-53.1427, *P*<0.01, vs sham operation group.

### The changes of NE in peripheral tissues of the hematoma

NE content in the peripheral tissues of the hemotoma varied little at the time points for measurement in the sham operation group (*P*>0.05). NE began to rise 0.5 h after intracerebral hemorrhage, significantly different from the contents in the sham operation group and measurement before the hemorrhage (*P*<0.01), with the peak level occurring 24 h after operation followed by gradual decrease, while till 72 h, NE content dropped to a level remarkably lower than those before hemorrhage and in the sham operation group (*P*<0.05) (Table 2).

**Table 2:** Changes of NE content in the peripheral tissues of the hematoma (n=5, ng/g)

Group	Before operation	After operation (h)					
		0.5	6	12	24	48	72

Sham operation	250.76 ± 5.36	247.80 ± 12.06	254.38 ± 10.09	253.86 ± 9.97	256.54 ± 4.39	247.94 ± 12.74	260.24 ± 13.61
Hemorrhage	251.86 ± 17.21	331.76 ± 30.07^b^^d^	405.94 ± 14.42^b^^d^	481.02 ± 21.57^b^^d^	555.18 ± 20.31^b^^d^	341.36 ± 42.97^b^^d^	230.30 ± 11.31^a^^c^

a t= 2.3446,*P*<0.05;

b t=4.3250-25.5062, *P*<0.01, vs the content before operation;

c t=1.8593, *P*<0.05;

d t=5.7941-32.1425,*P*<0.01, vs sham operation group.

### Changes of NE in the hypothalamus

Similarly, NE content did not vary significantly in the hypothalamus at the time points in the sham operation group (*P*>0.05). NE began to rise at 0.5 h after intracerebral hemorrhage to a level markedly higher than those of the sham operation group and before the hemorrhage (*P*<0.01), peaked 24 h after operation followed by gradual decrease, but till 72 h after the operation, its level was still higher than those before hemorrhage and in the sham operation group (*P*<0.01) (Table 3).

**Table 3:** Changes of NE in the rat hypothalamus after operation in the two groups (n=5, ng/g)

Group	Before operation	After operation (h)					
		0.5	6	12	24	48	72

Sham operation	3199.68 ± 170.55	3385.18 ± 241.99	3273.08 ± 163.93	3129.44 ± 197.18	3140.98 ± 194.51	3282.76 ± 63.144	3188.94 ± 231.47
Hemorrhage	3125.64 ± 142.84	3901.54 ± 215.02^a^^b^	4984.96 ± 456.27^a^^b^	5325.84 ± 574.16^a^^b^	7846.16 ± 416.12^a^^b^	5640 ± 774.49^a^^b^	3970.64 ± 250.37^a^^b^

a t=6.5549-23.9921, *P*<0.01, vs the level before operation;

b t=3.5666-22.9048, *P*<0.01, vs sham operation group.

### Changes of NE in the brainstem

Almost identical changes took place in NE content in the brain stem, as compared with its changes in the hypothalamus after operation (Table 4).

**Table 4:** Changes of NE in the brainstem of the rats in the two groups after operation (n=5, ng/g)

Group	Before operation	After operation (h)					
		0.5	6	12	24	48	72

Sham operation	376.32 ± 7.93	393.06 ± 17.70	370.58 ± 9.04	372.98 ± 17.11	378.18 ± 11.61	377.9 ± 7.50	371.48 ± 11.21
Hemorrhage	377.20 ± 10.72	430.28 ± 25.78^a^^b^	576.56 ± 15.56^a^^b^	740.18 ± 21.78^a^^b^	761.06 ± 15.52^a^^b^	706.50 ± 23.2^a^^b^	679.90 ± 22.93^a^^b^

a t= 7.8435-47.6938, *P*<0.01, vs the level before operation;

b t=6.6480-46.2482, *P*<0.01, vs sham operation group.

## DISCUSSIONS

The changes of brain monoamine transmitters in the event of cerebral injury and the secondary brain injury have drawn much attention. Studies demonstrate that exhaustion of the monoamine transmitters in the brain after cerebral infarction may reduce the volume of infarction, indicating the involvement of the monoamine transmitters in the pathogenesis and development of cerebral infarction (4). In another study, it was found that found that NE content was elevated in the serum and cerebrospinal fluid in most patients with acute cerebral blood deficiency, and NE was therefore believed to participate in the pathophysiology of injury to the blood-brain barrier following brain injury (5). In this present study, we found that the changes of NE in rat brain at various time points of cerebral hemorrhage followed the pattern that 30 min after cerebral hemorrhage, NE level was remarkably elevated in the peripheral tissues of the hematoma, hypothalamus and brainstem, peaked at 24 h, while during absorption period of the hematoma (48-72 h after hemorrhage), gradual decrease of NE took place. Although NE in the hypothalamus at 72 h was remarkable lower than its level at 24 h, it remained higher than that of the preoperative level, while NE in the peripheral tissues of the hematoma was lower than the preoperative level. At the same time, the neurobehavioral score varied synchronously together with NE.

Recent studies have identified energy metabolic disturbance and cessation of NE intake by the synaptosome as the major factors for extracellular NE transmitter retention (6). At the same time, lowered activities of aerobic enzymes, such as monoamine oxidase (MAO), occurs with reduced decomposition of monoamine into homovanillic acid (HVA) and 5-ketoindole acetic acid. In addition, due to excitement of the sympatho-adrenomedullary system after brain injury that triggers massive NE release into the blood stream, and the opening of the blood-brain barrier at the early stage of brain injury, NE in the blood may gain direct access into the cerebrum leading to increased NE content in the brain (7). While in the later period, NE is exhausted as a result of function loss of the nerve endings for NE intake and storage, and decreased activity of β-hydroxylase, which is especially obvious in the peripheral tissues of the hematoma.

Large amount of NE accumulated around the brain tissue, on the one hand promotes Ca^2+^ overload in the neural cells to cause delayed death of the cells (8), and on the other, leads to excessive neural cell excitement to accelerate energy consumption and lower the activities of many enzymes (such as Na-KATP enzyme) necessary for maintaining normal brain metabolism, resulting in abnormal ion distribution in the neural cells, which ultimately induce and exacerbates secondary injuries of the brain cells (9). Experiments indicate that NE may even act on α and β receptors in the vascular to cause intense contraction and spasm of the cerebral vessels, followed by disturbance of cerebrovascular contractions and brain microcirculation disorder, thus aggravating brain ischemia, edema and necrosis; in the meantime, large amount of NE may directly injure the endothelial cells of the brain vessels, leading to the leakage of the blood-brain barrier and aggravating cerebral edema (10). NE may stimulate the brain cells to secrete brain natriuretic peptide to result in hyponatremia (11), which is very likely an important mechanism contributing to remarkable increase of water content in the brain tissue and aggravating edema of the peripheral tissues of thehematoma.

It is therefore clear that large amount of NE, accumulated in the brain tissue in the course of cerebral hemorrhage, may hamper functional recovery of the brain. Prompt application of β-receptor agonists during the early stage of cerebral hemorrhage may be helpful for the management of brain injury.

## References

[1] Xu ZQ, Jiang XJ, Chen ME (2003). Alterations neuropeptide Y activity in brain tissue of rats during cerebral hemorrhage and its significance. Zhongguo Linchuang Kangfu.

[2] Longa EZ, Weinstein PR, Carson S (1989). Reversible middle cerebral artery occulusion without craniectomy in rats. Stroke.

[3] Luo LR, Liang YF, Yao YX (2002). The relationship between plasma level of monoamine neurohumor and attention deficit disorder with hypractivity and autistc disorder. Zhongguo Linchuang Kangfu.

[4] Nellgard B, Mackensen GB, Sarraf-Yazdi (1999). Pre-ischemic depletion of brain norepinephrine decreases infarct size in normothermic rats exposed to transient focal cerebral ischemia. Neurosci. Lett.

[5] Mautes AE, Muller M, Cortbus F (2001). Alterations of norepinephrine levels in plasma and CSF of patients after traumatic brain injury in relation to disruption of the blood-brain barrier. Acta. Neurochir. (Wien).

[6] Akiyama Y, Ito A, Koshimura K (1991). Effects of transient fore-brain ischemia and reperfusion on function of dopaminergic neurons and dopamine reuptakes *in vivo* in rat striatum. Brain Res.

[7] Dunn-Meynell AA, Hassanain M, Lovin BE (1998). Norepinephrine and traumatic brain injury: a possible role in post-trauma edema. Brain Res.

[8] Noh J, Kim EY, Kang JS (1999). Neurotoxic and neuroprotective actions of catecholamines in cortical neurons. Exp. Neurol.

[9] Kroppenstedt SN, Sakowitz OW, Thomale UW (2002). Influence of norepinephrine and dopamine on cortical perfusion, EEG activity, extracellular glutamate, and brain edema in rats after controlled cortical impact injury. J. Neurotrauma.

[10] Zhou AM, Zhang YH, Zhang LC (2002). The study of correlation between the nitrous oxide and nitrous oxide syntheses and brain infarction area. Zhongguo Linchuang Kangfu.

[11] Tomida M, Muraki M, Uemara K (1998). Plasma concentration of brain natriuretic peptide in patients with subarachniod hemorrhage. Stroke.

